# 2,2′-Dimethyl-2,2′-(*m*-phenyl­ene­dimethyl­ene)propane­dinitrile

**DOI:** 10.1107/S1600536809023344

**Published:** 2009-06-20

**Authors:** Junmei Zhao, Volker Böhmer, Michael Bolte

**Affiliations:** a Fachbereich Chemie, Pharmazie und Geowissenschaften, Abteilung Lehramt Chemie, Johannes Gutenberg-Universität Mainz, Duesbergweg 10-14, 55099 Mainz, Germany; b Institut für Anorganische Chemie, Johann Wolfgang Goethe-Universität Frankfurt, Max-von-Laue-Strasse 7, 60438 Frankfurt/Main, Germany

## Abstract

The title compound, C_16_H_14_N_4_, features an aromatic ring with two 2,2′-dicyano­propyl residues in positions 1 and 3, which are located above and below the ring plane. The two residues differ in their conformation with respect to the aromatic ring: whereas one of the C_meth­yl_—C—C_methyl­ene_—C_aromatic_ torsion angles is *gauche* [68.93 (12)°], the other one is fully staggered [177.63 (9)°]. The crystal structure is stabilized by C—H⋯N hydrogen-bonding inter­actions.

## Related literature

Calix[4]arenes, fixed in their cone conformation, offer a platform to attach various ligating functions *via* amide bonds to their wide or narrow rim, see: Arnaud-Neu *et al.* (1996 ▶); Barboso *et al.* (1999 ▶); Casnati *et al.* (2005 ▶); Danila *et al.* (2005*a* ▶,*b* ▶). Tetranitriles are suitable precursors for the attachment to the narrow rim. The title compound was envisaged as another potential tetranitrile. It is readily available by alkylation of methylmalonodinitrile with 1,3-bis-chloromethylbenzene.
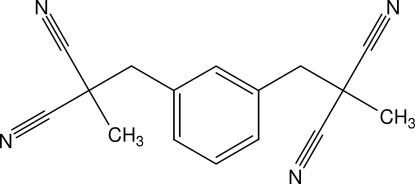


## Experimental

### 

#### Crystal data

C_16_H_14_N_4_
*M* *_r_* = 262.31
Monoclinic, 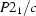

*a* = 11.6475 (10) Å
*b* = 13.3751 (13) Å
*c* = 9.4691 (9) Å
β = 99.020 (7)°
*V* = 1456.9 (2) Å^3^
*Z* = 4
Mo *K*α radiation
μ = 0.07 mm^−1^
*T* = 173 K
0.47 × 0.24 × 0.22 mm

#### Data collection

Stoe IPDS-II two-circle diffractometer
Absorption correction: none
8679 measured reflections
2725 independent reflections
2410 reflections with *I* > 2σ(*I*)
*R*_int_ = 0.038

#### Refinement

*R*[*F* ^2^ > 2σ(*F* ^2^)] = 0.037
*wR*(*F* ^2^) = 0.101
*S* = 1.04
2725 reflections
182 parameters
H-atom parameters constrained
Δρ_max_ = 0.22 e Å^−3^
Δρ_min_ = −0.16 e Å^−3^

### 

Data collection: *X-AREA* (Stoe & Cie, 2001 ▶); cell refinement: *X-AREA*; data reduction: *X-AREA*; program(s) used to solve structure: *SHELXS97* (Sheldrick, 2008 ▶); program(s) used to refine structure: *SHELXL97* (Sheldrick, 2008 ▶); molecular graphics: *XP* in *SHELXTL-Plus* (Sheldrick, 2008 ▶); software used to prepare material for publication: *SHELXL97*.

## Supplementary Material

Crystal structure: contains datablocks I, global. DOI: 10.1107/S1600536809023344/at2821sup1.cif

Structure factors: contains datablocks I. DOI: 10.1107/S1600536809023344/at2821Isup2.hkl

Additional supplementary materials:  crystallographic information; 3D view; checkCIF report

## Figures and Tables

**Table 1:** Hydrogen-bond geometry (Å, °)

*D*—H⋯*A*	*D*—H	H⋯*A*	*D*⋯*A*	*D*—H⋯*A*
C11—H11*A*⋯N24^i^	0.99	2.49	3.4028 (17)	153

Symmetry code: (i) 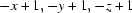.

**Fig. 1.**
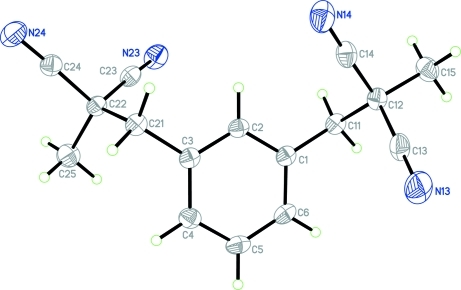
Perspective view of the title compound with the atom numbering; displacement ellipsoids are at the 50% probability level.

## supplementary crystallographic information

### Comment

Calix[4]arenes, fixed in their cone conformation, offer a platform to attach various ligating functions *via* amide bonds to their wide or narrow rim [*e.g.* CMPO-groups (Arnaud-Neu *et al.*, 1996; Barboso *et al.*, 1999), picolinamides (Casnati *et al.*, 2005)]. Suitable precursors for the attachment to the narrow rim are tetranitriles obtained by *O*-alkylation with ω-bromoalkyl nitriles, which can be easily reduced to the respective tetraamines (Danila *et al.*, 2005*a*,*b*). The title compound, 1,3-bis-(2,2'-dicyano)propyl benzene, (Fig. 1), was envisaged as another potential tetranitrile. It is readily available by alkylation of methylmalonodinitrile with 1,3-bis-chloromethylbenzene.

The title compound features an aromatic ring with two 2,2'-dicyano-propyl residues in positions 1 and 3, which are located above and below the ring plane. The two residues differ in their conformation with respect to the aromatic ring: whereas one of the C_methyl_-C-C_methylene_-C_aromatic_ torsion angles is gauche [68.93 (12)\%], the other one is fully staggered [177.63 (9)\%]. The crystal structure is stabilized by C—H···N hydrogen-bonding interactions (Table 1).

### Experimental

A solution of methylmalonodinitrile (1.59 g, 13.9 mmol) in dry acetone (10 ml) was added to a suspension of K_2_CO_3_ (3.95 g, 28.6 mmol) in 20 ml acetone. The mixture was stirred for 30 min. Then 1,3-bischloromethylbenzene (500 mg, 2.86 mmol) and KI (80.5 g) were added and the mixture was stirred 10 h under reflux. The solvent was evaporated to dryness and the residue taken up with CHCl_3_ (50 ml), washed with 1 *M* HCl, (2 *x* 30 ml), water and brine and dried over MgSO_4_. Evaporation of the solvent and purification by column chromatography (hexane/ethylacetate 5:1) gave 530 mg (71%) of 1,3-bis-(2,2'-dicyano)propyl benzene, m.p. 130–131°C; ^1^H NMR (400 MHz, CDCl_3_): δ / p.p.m.: 1.82 (s, 6H), 3.23 (s, 4H), 7.34 (d, 1H), 7.4–7.5 (m, 3H); MS(FD): m/*z* 262.3 (100%, *M*^+^).

Single crystals were obtained by slow evaporation of a solution in hexane/chloroform.

### Refinement

All H atoms could be located by difference Fourier synthesis. They were refined with fixed individual displacement parameters [U_iso_(H) = 1.2 *U*_eq_(C) or U_iso_(H) = 1.5 *U*_eq_(C_methyl_)] using a riding model with C_aromatic_—H = 0.95 Å, C_methyl_—H = 0.98Å and C_methylene_—H = 0.99 Å.

### Crystal data

**Table:**

C_16_H_14_N_4_	*F*(000) = 552
*M**_r_* = 262.31	*D*_x_ = 1.196 Mg m^−^^3^
Monoclinic, *P*2_1_/*c*	Mo *K*α radiation, λ = 0.71073 Å
Hall symbol: -P 2ybc	Cell parameters from 8902 reflections
*a* = 11.6475 (10) Å	θ = 3.5–25.7°
*b* = 13.3751 (13) Å	µ = 0.07 mm^−^^1^
*c* = 9.4691 (9) Å	*T* = 173 K
β = 99.020 (7)°	Block, colourless
*V* = 1456.9 (2) Å^3^	0.47 × 0.24 × 0.22 mm
*Z* = 4	

### Data collection

**Table:**

Stoe IPDS-II two-circle diffractometer	2410 reflections with *I* > 2σ(*I*)
Radiation source: fine-focus sealed tube	*R*_int_ = 0.038
graphite	θ_max_ = 25.6°, θ_min_ = 3.5°
ω scans	*h* = −14→14
8679 measured reflections	*k* = −16→16
2725 independent reflections	*l* = −11→8

### Refinement

**Table:**

Refinement on *F*^2^	Secondary atom site location: difference Fourier map
Least-squares matrix: full	Hydrogen site location: inferred from neighbouring sites
*R*[*F*^2^ > 2σ(*F*^2^)] = 0.037	H-atom parameters constrained
*wR*(*F*^2^) = 0.101	*w* = 1/[σ^2^(*F*_o_^2^) + (0.0522*P*)^2^ + 0.3439*P*] where *P* = (*F*_o_^2^ + 2*F*_c_^2^)/3
*S* = 1.03	(Δ/σ)_max_ < 0.001
2725 reflections	Δρ_max_ = 0.22 e Å^−^^3^
182 parameters	Δρ_min_ = −0.16 e Å^−^^3^
0 restraints	Extinction correction: *SHELXL97* (Sheldrick, 2008), Fc^*^=kFc[1+0.001xFc^2^λ^3^/sin(2θ)]^-1/4^
Primary atom site location: structure-invariant direct methods	Extinction coefficient: 0.035 (4)

### Special details

**Table:**

Geometry. All e.s.d.'s (except the e.s.d. in the dihedral angle between two l.s. planes) are estimated using the full covariance matrix. The cell e.s.d.'s are taken into account individually in the estimation of e.s.d.'s in distances, angles and torsion angles; correlations between e.s.d.'s in cell parameters are only used when they are defined by crystal symmetry. An approximate (isotropic) treatment of cell e.s.d.'s is used for estimating e.s.d.'s involving l.s. planes.
Refinement. Refinement of *F*^2^ against ALL reflections. The weighted *R*-factor *wR* and goodness of fit *S* are based on *F*^2^, conventional *R*-factors *R* are based on *F*, with *F* set to zero for negative *F*^2^. The threshold expression of *F*^2^ > σ(*F*^2^) is used only for calculating *R*-factors(gt) *etc*. and is not relevant to the choice of reflections for refinement. *R*-factors based on *F*^2^ are statistically about twice as large as those based on *F*, and *R*- factors based on ALL data will be even larger.

### Fractional atomic coordinates and isotropic or equivalent isotropic displacement parameters (Å^2^)

**Table:**

	*x*	*y*	*z*	*U*_iso_*/*U*_eq_	
C1	0.24600 (9)	0.73229 (9)	0.32550 (12)	0.0222 (3)	
C2	0.32892 (9)	0.68926 (8)	0.43145 (12)	0.0225 (3)	
H2	0.3344	0.6185	0.4382	0.027*	
C3	0.40374 (9)	0.74802 (8)	0.52747 (12)	0.0217 (3)	
C4	0.39613 (10)	0.85248 (9)	0.51626 (12)	0.0256 (3)	
H4	0.4461	0.8935	0.5809	0.031*	
C5	0.31502 (10)	0.89599 (9)	0.41008 (13)	0.0285 (3)	
H5	0.3107	0.9667	0.4019	0.034*	
C6	0.24024 (10)	0.83659 (9)	0.31583 (12)	0.0262 (3)	
H6	0.1849	0.8672	0.2445	0.031*	
C11	0.16383 (10)	0.66726 (9)	0.22412 (12)	0.0242 (3)	
H11A	0.2093	0.6129	0.1883	0.029*	
H11B	0.1300	0.7083	0.1409	0.029*	
C12	0.06227 (10)	0.61940 (9)	0.29147 (12)	0.0240 (3)	
C13	−0.00481 (10)	0.69976 (10)	0.35020 (14)	0.0313 (3)	
N13	−0.05588 (11)	0.76326 (11)	0.39209 (16)	0.0516 (4)	
C14	0.10897 (10)	0.55154 (10)	0.41158 (13)	0.0280 (3)	
N14	0.14425 (10)	0.49664 (10)	0.50128 (13)	0.0423 (3)	
C15	−0.01964 (11)	0.55815 (10)	0.17831 (13)	0.0328 (3)	
H15A	0.0249	0.5053	0.1399	0.049*	
H15B	−0.0539	0.6024	0.1005	0.049*	
H15C	−0.0817	0.5280	0.2231	0.049*	
C21	0.48821 (9)	0.69770 (9)	0.64449 (12)	0.0233 (3)	
H21A	0.4519	0.6357	0.6740	0.028*	
H21B	0.5017	0.7426	0.7285	0.028*	
C22	0.60898 (9)	0.67039 (9)	0.59992 (12)	0.0227 (3)	
C23	0.59040 (10)	0.60957 (9)	0.46639 (12)	0.0262 (3)	
N23	0.57367 (10)	0.56598 (9)	0.36058 (12)	0.0371 (3)	
C24	0.67231 (10)	0.60763 (9)	0.71679 (12)	0.0256 (3)	
N24	0.71923 (10)	0.56270 (8)	0.81204 (12)	0.0376 (3)	
C25	0.68272 (11)	0.76360 (10)	0.57854 (14)	0.0325 (3)	
H25A	0.6947	0.8033	0.6666	0.049*	
H25B	0.6420	0.8042	0.5001	0.049*	
H25C	0.7582	0.7425	0.5554	0.049*	

### Atomic displacement parameters (Å^2^)

**Table:**

	*U*^11^	*U*^22^	*U*^33^	*U*^12^	*U*^13^	*U*^23^
C1	0.0200 (5)	0.0253 (6)	0.0232 (5)	0.0021 (4)	0.0095 (4)	0.0013 (4)
C2	0.0205 (5)	0.0209 (5)	0.0280 (6)	0.0022 (4)	0.0095 (4)	0.0023 (4)
C3	0.0190 (5)	0.0245 (6)	0.0236 (6)	0.0023 (4)	0.0100 (4)	0.0006 (4)
C4	0.0249 (6)	0.0243 (6)	0.0293 (6)	0.0005 (5)	0.0090 (5)	−0.0036 (5)
C5	0.0318 (6)	0.0207 (6)	0.0350 (6)	0.0053 (5)	0.0112 (5)	0.0014 (5)
C6	0.0257 (6)	0.0261 (6)	0.0280 (6)	0.0075 (5)	0.0083 (5)	0.0049 (5)
C11	0.0245 (5)	0.0275 (6)	0.0214 (5)	0.0026 (5)	0.0064 (4)	0.0009 (4)
C12	0.0224 (5)	0.0279 (6)	0.0218 (6)	0.0015 (5)	0.0042 (4)	−0.0024 (4)
C13	0.0207 (5)	0.0397 (7)	0.0335 (6)	−0.0011 (5)	0.0047 (5)	−0.0086 (5)
N13	0.0343 (6)	0.0557 (8)	0.0666 (9)	0.0067 (6)	0.0131 (6)	−0.0225 (7)
C14	0.0217 (5)	0.0377 (7)	0.0258 (6)	−0.0038 (5)	0.0071 (5)	0.0008 (5)
N14	0.0298 (6)	0.0580 (8)	0.0390 (6)	−0.0046 (5)	0.0051 (5)	0.0170 (6)
C15	0.0347 (6)	0.0351 (7)	0.0268 (6)	−0.0059 (5)	−0.0005 (5)	−0.0038 (5)
C21	0.0225 (5)	0.0265 (6)	0.0224 (5)	0.0015 (4)	0.0077 (4)	0.0004 (4)
C22	0.0216 (5)	0.0239 (6)	0.0233 (5)	0.0021 (4)	0.0056 (4)	0.0013 (4)
C23	0.0249 (6)	0.0286 (6)	0.0259 (6)	0.0067 (5)	0.0064 (5)	0.0034 (5)
N23	0.0407 (6)	0.0402 (6)	0.0303 (6)	0.0102 (5)	0.0054 (5)	−0.0049 (5)
C24	0.0257 (6)	0.0251 (6)	0.0265 (6)	0.0031 (5)	0.0056 (5)	−0.0025 (5)
N24	0.0444 (7)	0.0345 (6)	0.0323 (6)	0.0110 (5)	0.0011 (5)	0.0017 (5)
C25	0.0271 (6)	0.0327 (7)	0.0391 (7)	−0.0043 (5)	0.0096 (5)	0.0042 (5)

### Geometric parameters (Å, °)

**Table:**

C1—C6	1.3989 (16)	C12—C15	1.5521 (16)
C1—C2	1.4024 (16)	C13—N13	1.1431 (18)
C1—C11	1.5185 (16)	C14—N14	1.1489 (17)
C2—C3	1.3983 (16)	C15—H15A	0.9800
C2—H2	0.9500	C15—H15B	0.9800
C3—C4	1.4029 (16)	C15—H15C	0.9800
C3—C21	1.5189 (15)	C21—C22	1.5737 (15)
C4—C5	1.3944 (17)	C21—H21A	0.9900
C4—H4	0.9500	C21—H21B	0.9900
C5—C6	1.3938 (17)	C22—C24	1.4891 (16)
C5—H5	0.9500	C22—C23	1.4905 (16)
C6—H6	0.9500	C22—C25	1.5452 (16)
C11—C12	1.5661 (16)	C23—N23	1.1491 (16)
C11—H11A	0.9900	C24—N24	1.1483 (16)
C11—H11B	0.9900	C25—H25A	0.9800
C12—C13	1.4870 (17)	C25—H25B	0.9800
C12—C14	1.4898 (16)	C25—H25C	0.9800
			
C6—C1—C2	118.46 (11)	C15—C12—C11	110.88 (9)
C6—C1—C11	120.72 (10)	N13—C13—C12	178.01 (15)
C2—C1—C11	120.82 (10)	N14—C14—C12	177.80 (14)
C3—C2—C1	121.57 (10)	C12—C15—H15A	109.5
C3—C2—H2	119.2	C12—C15—H15B	109.5
C1—C2—H2	119.2	H15A—C15—H15B	109.5
C2—C3—C4	119.01 (10)	C12—C15—H15C	109.5
C2—C3—C21	119.45 (10)	H15A—C15—H15C	109.5
C4—C3—C21	121.50 (10)	H15B—C15—H15C	109.5
C5—C4—C3	119.86 (11)	C3—C21—C22	114.28 (9)
C5—C4—H4	120.1	C3—C21—H21A	108.7
C3—C4—H4	120.1	C22—C21—H21A	108.7
C6—C5—C4	120.56 (11)	C3—C21—H21B	108.7
C6—C5—H5	119.7	C22—C21—H21B	108.7
C4—C5—H5	119.7	H21A—C21—H21B	107.6
C5—C6—C1	120.52 (11)	C24—C22—C23	108.18 (9)
C5—C6—H6	119.7	C24—C22—C25	109.35 (9)
C1—C6—H6	119.7	C23—C22—C25	109.81 (10)
C1—C11—C12	114.71 (9)	C24—C22—C21	106.92 (9)
C1—C11—H11A	108.6	C23—C22—C21	109.70 (9)
C12—C11—H11A	108.6	C25—C22—C21	112.74 (9)
C1—C11—H11B	108.6	N23—C23—C22	177.17 (12)
C12—C11—H11B	108.6	N24—C24—C22	176.36 (12)
H11A—C11—H11B	107.6	C22—C25—H25A	109.5
C13—C12—C14	107.77 (10)	C22—C25—H25B	109.5
C13—C12—C15	109.73 (10)	H25A—C25—H25B	109.5
C14—C12—C15	108.47 (10)	C22—C25—H25C	109.5
C13—C12—C11	109.36 (10)	H25A—C25—H25C	109.5
C14—C12—C11	110.56 (9)	H25B—C25—H25C	109.5
			
C6—C1—C2—C3	0.90 (16)	C6—C1—C11—C12	−104.02 (12)
C11—C1—C2—C3	−178.74 (10)	C2—C1—C11—C12	75.62 (13)
C1—C2—C3—C4	−0.63 (16)	C1—C11—C12—C13	56.49 (13)
C1—C2—C3—C21	177.21 (10)	C1—C11—C12—C14	−62.02 (13)
C2—C3—C4—C5	−0.23 (16)	C1—C11—C12—C15	177.63 (9)
C21—C3—C4—C5	−178.02 (10)	C2—C3—C21—C22	88.93 (12)
C3—C4—C5—C6	0.80 (17)	C4—C3—C21—C22	−93.29 (12)
C4—C5—C6—C1	−0.52 (17)	C3—C21—C22—C24	−170.87 (9)
C2—C1—C6—C5	−0.32 (17)	C3—C21—C22—C23	−53.79 (13)
C11—C1—C6—C5	179.32 (10)	C3—C21—C22—C25	68.93 (12)

### Hydrogen-bond geometry (Å, °)

**Table:**

*D*—H···*A*	*D*—H	H···*A*	*D*···*A*	*D*—H···*A*
C11—H11A···N24^i^	0.99	2.49	3.4028 (17)	153

Symmetry codes: (i) −*x*+1, −*y*+1, −*z*+1.
